# The effect of serum cortisol level on the outcomes of Persistent Inflammation, Immunosuppression and Catabolism Syndrome patients in the intensive care unit

**DOI:** 10.12669/pjms.41.2.10256

**Published:** 2025-02

**Authors:** Onur Eroglu, Asu Ozgultekin, Osman Ekinci

**Affiliations:** 1 Onur Eroglu Department of Anesthesiology and Intensive Care, Susehri State Hospital, 58600 Sivas, Turkey; 2 Asu Ozgultekin Associate Professor, Department of Anesthesiology and Intensive Care, Haydarpasa Numune Training and Research Hospital, University of Health Sciences, 34674 Istanbul, Turkey; 3 Osman Ekinci Professor, Department of Anesthesiology and Intensive Care, Haydarpasa Numune Training and Research Hospital, University of Health Sciences, 34674 Istanbul, Turkey

**Keywords:** Adrenocortical insufficiencies, Intensive care unit, Persistent inflammation immunosuppression catabolism syndrome

## Abstract

**Objective:**

This study sought to elucidate the potential association between serum cortisol level and clinical outcomes in patients diagnosed with Persistent Inflammation, Immunosuppression, and Catabolism Syndrome (PIICS).

**Methods:**

This prospective observational study, initiated in January 2023 and concluded in July 2023 enrolled 42 patients diagnosed with PIICS admitted to the intensive care unit (ICU) at Training and Research Hospital. For the purpose of analysis, serum cortisol levels were categorized as low (<15 μg/dL) and high (>15 μdL). To facilitate data organization and subsequent analysis, measurements were categorized into three time intervals following ICU admission: T1 (days 14-21), T2 (days 21-28), and T3 (days >28). Statistical analysis was performed using IBM-SPSS 28. A significance level of p < 0.05 was set to determine statistically significant differences between groups.

**Results:**

Cortisol measured at T3 were significantly lower (p < 0.05) compared to T2. In contrast, SOFA scores were significantly higher (p < 0.05). No statistically significant differences were observed between the low and high cortisol level groups at T1, T2 and T3 in terms of gender, APACHE-2 score, SOFA score, ICU length of stay (days), duration of mechanical ventilation (days), mortality rate, or mechanical ventilation requirement at discharge. Patients in the low cortisol group at T3 exhibited a significantly higher mean age compared to those in the high cortisol group (p < 0.05).

**Conclusion:**

Cortisol levels in ICU patients may change over time. That cortisol levels tend to decrease as the length of stay increases, in older patients, and in PIICS patients with elevated SOFA scores.

## INTRODUCTION

Early deaths from many diseases have now been prevented. However, many people who have survived acute critical illness experience a condition called persistent inflammation, immunosuppression and catabolism syndrome (PIICS).^1^ The increased number of days of ICU stay in these patients, the need for follow-up in special centers after ICU, and the high costs due to recurrent hospitalizations have led to the need to review this group in terms of risk factors.^2^

Several studies suggest the potential benefit of serially monitoring cortisol levels in patients with prolonged lengths of stay within the ICU.^3^ Adrenocortical insufficiency has been defined in cases of chronic critical illness (CCI) with ICU stay for more than 14 days and organ dysfunctions.^4^ Lenght of stay in the ICU was found to be longer in patients with low cortisol levels.^3^,^5^

Timely identification and management of adrenocortical insufficiency in patients with CCI have the potential to expedite ICU discharge and optimize resource allocation. In light of the association between adrenocortical insufficiency and prolonged ICU stay in CCI patients, this study sought to investigate the relationship between cortisol levels and clinical outcomes in a specific subgroup of CCI: PIICS.

## METHODS

This study enrolled patients admitted to the ICU of Training and Research Hospital, minimum duration of 14 days, with confirmation of at least one serum cortisol measurement during their ICU stay. The study was a prospective observational cohort study initiated in January 2023 and concluded in July 2023. Sample size analysis was performed using a 95% confidence interval and 80% power, which suggested that at least 40 patients study was conducted with 42 patient.

### Ethical approval:

It was obtained from the Medical Ethics Committee of Training and Research Hospital (decision number HNHAH-KAEK2022/225, December 2022).

A total of 137 patients admitted to the ICU between January and July 2023 were initially screened for participation in this study. Following application of exclusion criteria, 42 patients were ultimately enrolled.

### Exclusion Criteria:

Patients were excluded if they were under 18 years of age, had received corticosteroid therapy within the preceding month prior to cortisol measurement, or did not fulfill the established criteria for PIICS diagnosis. Patients continued to receive treatment for their underlying illness diagnoses and standard ICU management protocols as determined by their attending intensivist.

The intensivist decides whether to test a patient for serum cortisol based on clinical features suggestive of corticosteroid insufficiency. Peripheral venous blood samples were collected at 08:00a.m. Serum cortisol concentrations were quantified using the IMMULITE® 2000 assay (Diagnostic Products Corporation, Los Angeles,USA). Demographic information and primary diagnoses leading to ICU admission were documented for all participants. During hospitalization, comprehensive clinical data were collected for all participants, including Acute Physiology and Chronic Health Evaluation (APACHE)II score and Sequential Organ Failure Assessment (SOFA) scales, serum albumin levels, C-Reactive Protein (CRP) levels, complete blood count, cortisol. For the purpose of analysis, serum cortisol levels were categorized as low (<15 μg/dL) and high (>15 μg/dL). To facilitate data organization and subsequent analysis, measurements were categorized into three time intervals following ICU admission: T1 (days 14-21), T2 (days 21-28), and T3 (days >28). Patients were classified as PIICS+ if they fulfilled all the following criteria at each time point of measurement derived from routine laboratory investigations: CRP exceeding 150 μg/dl, total lymphocyte count below 800 cells/mm3, and serum albumin less than three g/dl.

### Statistical analysis:

It was performed using IBM-SPSS Statistics software (version 28.0, IBM Corp. New York, USA). Mean, standard deviation (SD), median, lowest, highest, frequency, ratio values were used in the descriptive statistics of the data. Distribution of variables was measured with Kolmogorov Smirnov test. Independent sample t-test and Mann-Whitney U test were used in the analysis of quantitative independent data. Paired sample t test and Wilcoxon test were used in the analysis of dependent quantitative data. Chi-square test was used in the analysis of qualitative independent data, and Fischer test was used when chi-square test conditions were not met. A significance level of p < 0.05 was set to determine statistically significant differences between groups.

### Consent:

All procedures performed in the studies involving human participants were in accordance with the ethical standards of the institutional and/or national research committee and with the 1964 Helsinki Declaration and its later amendments or comparable ethical standards.

## RESULTS

Among the 137 patients initially screened, 95 did not meet the inclusion criteria and were excluded from further participation. This resulted in a final study population of 42 patients. The study population exhibited a wide age range, with participant ages spanning from 45 to 99 years old. The mean age was 75.93 (SD±13.247) years. The study population included 59.5% (n=25) male and 40.5% (n=17) famale. Pneumonia (28.6% n=12) and sepsis (19.1% n=8) were the leading causes of ICU admission.

The mortality rate was 66.7%, with 28 of the 42 participants succumbing. Among the 14 patients who were discharged from the ICU, seven required ongoing mechanical ventilation support. The study observed a mean length of stay in the ICU of 38.4 days (SD±24.7) mean duration of ventilation was 31.1 days (SD±25.5). Table-I presents cortisol levels and SOFA scores stratified by measurement time. Cortisol measured at T3 were significantly lower (p < 0.05) compared to T2. In contrast, SOFA scores were significantly higher (p < 0.05) at T3 compared to T2 (Table-I).

**Table-I:** Changes in SOFA Scores and Cortisol Values Over Measurement Time.

		T1	T2	T3	p	p*	p**	
SOFA Score	Mean±SD	5.0 ± 2.4	4.8 ± 2.2	5.4 ± 3.1	0.914	0.228	0.041	^W^
	Median	5.0	5.0	6.0				
Cortizol	Mean±SD	20.6 ± 10.6	20.9 ± 8.1	16.6 ± 5.2	0.092	0.906	**0.048**	^W^
	Median	18.2	20.2	15.3				

w Wilcoxon test, p T1-T2 Change, p* T1-T3 Change, p** T2-T3 Change, sd:Standard deviation

Cortisol levels were assessed at three time points among participants. At T1, high cortisol levels were observed in 30 of the 42 patients, while 12 had low levels. Trends were noted at T2 (21 high, eight low of 29 patients) and T3 (nine high, nine low of 18 patients). No statistically significant differences were observed between the low and high cortisol level groups at T1, T2 and T3 in terms of gender, APACHE-2 score, SOFA score, ICU length of stay (days), duration of mechanical ventilation (days), mortality rate, mechanical ventilation requirement at discharge (p>0.05)(Table-II, III and IV). Patients in the low cortisol group at T3 exhibited a significantly higher mean age compared to those in the high cortisol group (p < 0.05) (Table-IV). All three measurement times, mortality rates were higher in the low cortisol group, although this finding did not reach statistical significance (p > 0.05).

**Table-II:** Comparison of patients with low and high cortisol levels at the time of T1 measurement.

		T1 Cortizol <15		T1 Cortizol >15		p

		Mean±SD/n-%	Median	Mean±sd/n-%	Median	
Age		77.2 ± 15.3	82.0	75.4 ± 12.6	76.5	0.707 ^t^
Sex	Female	3 25.0%		14 46.7%		0.196 ^X²^
	Male	9 75.0%		16 53.3%	
Apache-II Score		21.5 ± 5.2	19.5	22.1 ± 6.0	21.0	0.775 ^t^
SOFA Score		5.9 ± 3.0	5.5	4.7 ± 2.0	4.0	0.266 ^m^
MV Duration (Days)		23.3± 8.7	21.0	34.2 ± 29.3	26.5	0.329 ^m^
Length of Stay (Days)		30.6 ± 9.7	30.0	41.5 ± 28.1	30.0	0.460 ^m^
Alive/Dead		Alive 4	33.3%	10	33.3%	1.000 ^X²^
		Dead 8	66.7%	20	66.7%
MV Requirement at Discharge		(-) 3	75.0%	4	40.0%	0.237 ^X²^
		(+) 1	25.0%	6	50.0%

**Table-III:** Comparison of patients with low and high cortisol levels at the time of T2 measurement.

		T2 Cortizol < 15		T2 Cortizol > 15			p

		Mean±sd/n-%	Median	Mean±sd/n-%	Median		
Age		81.0 ± 8.2	84.5	74.9 ± 12.1	74.0		0.201
Sex	Female	2 25.0%		9 42.9%		0.376 ^X²^	
	Male	6 75.0%		12 57.1%	
Apache-II Score		24.5 ± 5.8	23.5	21.6 ± 5.4	21.0		0.220 ^t^
SOFA Score		5.0 ± 3.0	5.0	4.7 ± 1.9	4.0		0.624 ^m^
MV Duration (Days)		43.8 ± 42.9	34.0	34.0 ± 21.5	28.0		0.678 ^m^
Length of Stay (Days)		52.0 ± 40.6	36.0	42.3 ± 20.3	33.0		0.845 ^m^
Alive/Dead	Alive	1 12.5%		8 38.1%		0.183 ^X²f^	
	Dead	7 87.5%		13 61.9%			
MV Requirement at Discharge	(-)	1 100.0%		3 37.5%		0.444 ^X²f^	
	(+)	0 0.0%		5 62.5%			

^t^Independent sample t-test / ^X²^ Chi-Square test / ^m^Mann-whitney u test / ^X²f^Chi-Square test (Fisher) / sd/n-% standard deviation / MV:Mechanical Ventilation.

**Table-IV:** Comparison of patients with low and high cortisol levels at the time of T3 measurement.

		T3 Cortizol <15		T3 Cortizol >15		p

		Mean±sd/n-%	Median	Mean±sd/n-%	Median	
Age		84.1 ± 10.2	86.0	69.2 ± 8.4	68.0	0.004 ^t^
Sex	Female	4 44.4%		2 22.2%		0.317 ^X²^
	Male	5 55.6%		7 77.8%	
Apache-II Score		24.8 ± 6.6	26.0	21.6 ± 5.8	22.0	0.287 ^t^
SOFA Score		5.6 ± 2.9	6.0	5.2 ± 3.5	4.0	0.530 ^m^
MV Duration (Days)		43.8 ± 41.0	38.0	45.3 ± 26.2	36.0	0.627 ^m^
Length of Stay (Days)		55.1 ± 36.8	52.0	54.0 ± 23.2	40.0	0.536 ^m^
Alive/Dead		Taburcu 2	22.2%	6	66.7%	0.058 ^X²^
		Ex 7	77.8%	3	33.3%
MV Requirement at Discharge		(-) 2	100.0%	2	33.3%	0.429 ^X²^
		(+) 0	0.0%	4	66.7%

## DISCUSSION

We found that, cortisol levels measured at T3 were significantly lower (p < 0.05) compared to T2. In contrast, SOFA scores were significantly higher at T3 (p < 0.05). There was no significant difference in mechanical ventilation duration, intensive care unit stay, mortality rate, and mechanical ventilation requirement at discharge between groups with low and high cortisol levels at T1, T2, and T3. While mortality rates were higher in the low cortisol group at all of the three measurement times, this finding did not reach statistical significance (p > 0.05). Our findings shows that in our group of CCI patients with PIICS average cortisol levels decreased over time, while SOFA scores, indicative of organ dysfunction, conversely increased.

There is widespread perception among clinicians that sepsis poses a significant therapeutic challenge.^6^ Despite this, earlier diagnosis and better compliance with Sepsis Survival Campaign resuscitation protocols are seen, facilitated by various biomarkers.^7^,^8^ This results in a decrease in mortality rates associated with diseases that cause MOF, including sepsis.^9^-^12^ Survivors of this critical illness may exhibit two distinct post-acute outcomes: a return to immunological homeostasis or persistent immunological dysfunction. This persistent immunological dysfunction can lead to the development of CCI, characterized by an ICU stay exceeding 14 days and the presence of lasting organ dysfunction. Approximately 30-50% of these patients experience a condition called PIICS.^13^ PIICS is predicted to be a substitute phenotype of late MOF and is often associated with poor clinical outcomes, prolonged ICU stay, increased costs and recurrent hospitalizations.^14^,^15^ Our study aimed to explore the role of cortisol levels in PIICS patients, with the goal of improving patient outcomes.

A prolonged ICU stay is associated with an increased risk of adrenocortical suppression.^16^ Some studies show that in CCI patients with normal initial cortisol levels, there is a decrease in cortisol levels in repeated measurements and the relationship of this decrease with survival.^3^,^5^ Talan et al. reported a significantly longer length of ICU stay in patients with low cortisol levels who recurrent vasopressor needs CCI patients and there is no need for vasopressor after replacement^5^ Our study did not observe a statistically significant difference in length of stay between patients with high and low cortisol levels at any of the measurement times.

Although it did not reach statistical significance, mortality rates were higher in the low cortisol group at all three measurement times (p > 0.05). This difference may be attributable to the distinct study populations. Our investigation focused on patients with PIICS, whereas other studies might have included broader CCI populations. Four patients within the low cortisol group received corticosteroid replacement, as determined by the attending intensivist. Three of these patients succumbed to their condition, while one patient was discharged. Our observation in PIICS patients lays the groundwork for future, well designed controlled trials to definitively assess the effectiveness of corticosteroid replacement therapy in this patient population. Thus it’s aims to contribute to the literature by providing new insights into the management of critically ill patients, particularly those with PIICS.

The suppressed adrenocortical function in ICU patients, serum cortisol levels increased initially due to a decrease in cortisol degradation and binding proteins. The level may appear high.^17^-^19^ When the ICU stay is prolonged, there is a decrease in serum cortisol level, especially after the 28th day. This condition is a chronic process associated with the patient’s prolonged hospitalization.^17^ According to Wu et al., cortisol levels decreased compared to baseline in subsequent measurements in CCI patients.^3^ Similarly, in our study, cortisol value at T3 time (after the 28th day) showed a significant decrease compared to T2 time.

In our study, the average age of the group with low cortisol levels was higher at all three measurement times. This situation has statistical significance in the measurement at T3 time (p<0.05). We can interpret this result as elderly patients, whose organ capacity is already limited, experiencing chronic and permanent critical illness with more damage. SOFA scoring may help evaluate organ dysfunction and predict outcomes. Both mean and peak SOFA scores are useful predictors.^20^ In Wu et al. study, there was no significant difference in SOFA scores between groups with low and high cortisol levels.^3^ In our study group, the T3 SOFA score increased significantly (p<0.05) compared to the T2 time. The decrease in cortisol level and the increase in SOFA score between measurements at T2 and T3 times suggest that there may be an inverse correlation between cortisol level and clinical outcomes (organ failure).

In CCI patients with PIICS average cortisol levels may decrease over time, while SOFA scores, indicative of organ dysfunction, conversely increase. This finding suggests a potential link between cortisol levels and organ failure. We hypothesize that the observed decrease in cortisol levels may be attributable to adrenocortical suppression secondary to chronic inflammation and recurrent infections. Our findings may pave the way for future studies to determine a threshold cortisol level in these patients and investigate the causal relationship between cortisol replacement therapy and patient outcomes.

### Limitations:

Limitation of the study is that the sample was a small group limited to intensive care patients in a single center. As an observational study, it cannot establish causality between cortisol levels and patient outcomes in the ICU. Several confounding variables, beyond the scope of this investigation, may have influenced patient outcomes. Additionally, physician discretion determined cortisol level measurement, potentially introducing selection bias. Furthermore, the lack of baseline cortisol data upon ICU admission limits the ability to assess changes from a patient’s individual norm.

## CONCLUSION

The findings suggest a time dependent decline in cortisol levels, particularly after the fourth week of ICU stay (T3). Additionally, lower cortisol levels were observed in older patients and those exhibiting greater organ dysfunction as measured by SOFA scores. There may be an inverse correlation between cortisol level and clinical outcomes (organ failure). We propose investigating the utility of monitoring serum cortisol levels in PIICS patients to identify potential cortisol deficiencies and assess the efficacy of cortisol replacement therapy in improving patient outcomes. We did not measure baseline cortisol level upon ICU admission, which hinders a comprehensive interpretation of the results.

### Reccomandations:

Future studies should include the measurement and recording of baseline cortisol levels to better understand the impact cortisol levels on the PIICS patients outcomes. Additionally, it is essential to record the treatment strategies employed for cortisol deficiency and to evaluate the response of these patients to replacement therapy as a subgroup analysis.

### Authors Contribution:

**OEU:** Designed and did statistical analysis & editing of manuscript, is responsible for integrity of research, data collection and manuscript writing.

**AO** and **OEI:** Did designed statistical analysis & editing of manuscript and did review and final approval of manuscript.
